# Transcatheter Intervention for Heart Failure: Excitement, Progress, and Trepidation

**DOI:** 10.1016/j.jscai.2023.101214

**Published:** 2023-12-04

**Authors:** Philippe Généreux, Jeffrey M. Testani

**Affiliations:** a Gagnon Cardiovascular Institute, Morristown Medical Center, Morristown, New Jersey; b Department of Internal Medicine, Section of Cardiovascular Medicine, Yale University School of Medicine, New Haven, Connecticut

**Keywords:** cardiorenal syndrome, device, heart failure, medical therapy, transcatheter

Heart failure is a prevalent disease associated with significant mortality and morbidity, reduced quality of life, and increased health care costs. Moreover, heart failure is one of the most common reasons for hospitalization and is the final common pathway of the majority of cardiac diseases, such as coronary artery disease, valvular disease, primary or secondary myocardial disease, and congenital anomaly.

**Figure 1:**
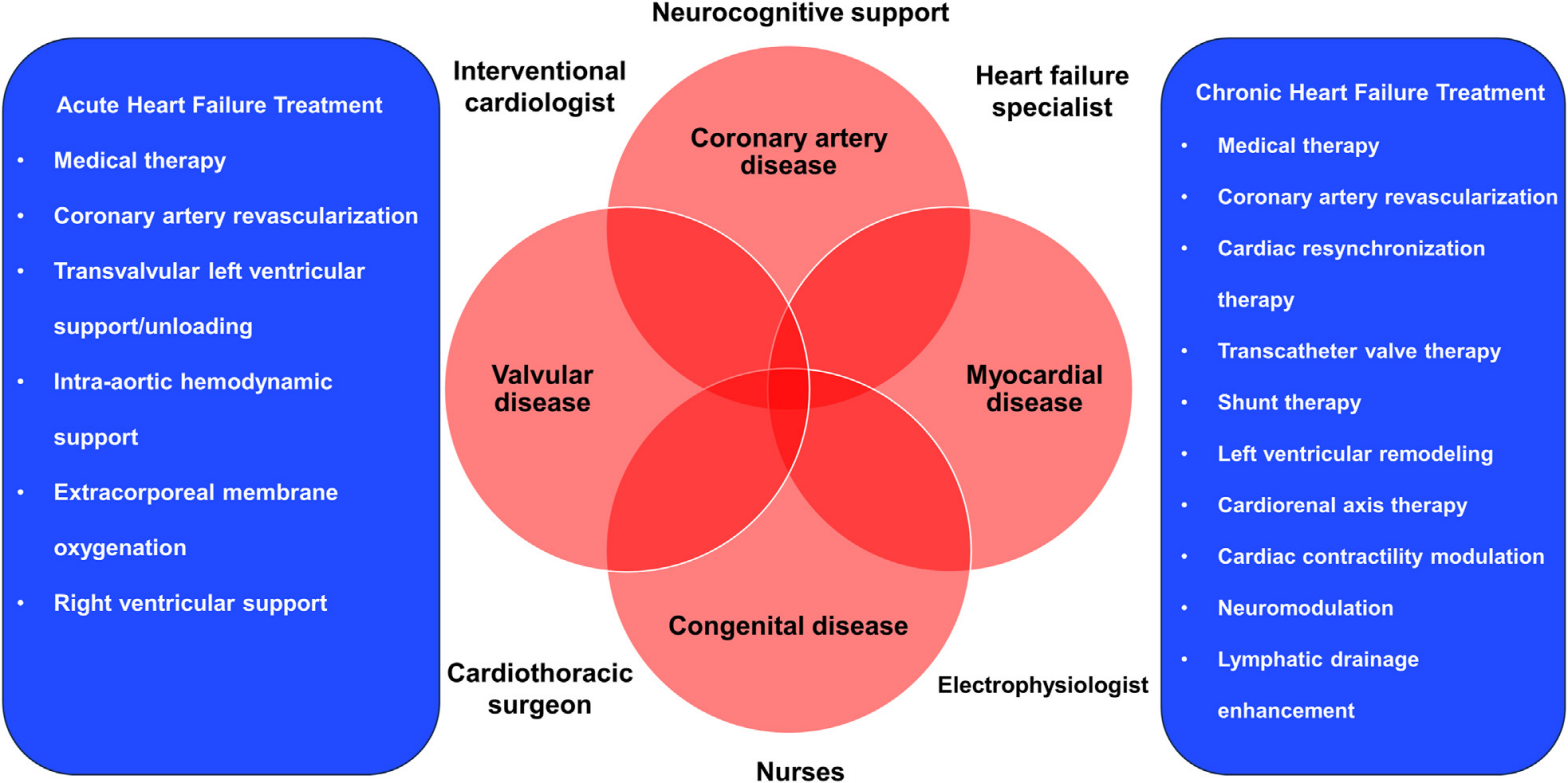
**Transcatheter interventions for heart failure.** Acute and chronic heart failure is common and originates from multiple causes. Although medical therapy, coronary revascularization, and cardiac resynchronization represent the first line of treatment, novel transcatheter options are currently under investigation and could potentially improve patient outcomes. Collaboration between cardiovascular specialists and health care providers is required to ensure optimal care of this complex population.

Contemporary treatment of heart failure has consisted primarily of medical therapy, cardiac resynchronization therapy, coronary revascularization, and surgical intervention when indicated. Efforts have been made to offer percutaneous options for patients presenting with acutely decompensated heart failure and cardiogenic shock requiring hemodynamic support^1^^,^^2^ or necessitating assistance in the context of complex or high-risk coronary revascularization.^3^ Over the past 2 decades, the field of percutaneous intervention for heart failure has evolved, in large part resulting from the development and expansion of transcatheter aortic valve replacement,^4^^,^^5^ followed by transcatheter edge-to-edge repair for the mitral and tricuspid valves.^6^^,^^7^ Recently, the emphasis has been on the development of therapies for chronic heart. Although some strategies intuitively focus on enhancing cardiac function by unloading or supporting the heart or mechanically improving left ventricular remodeling, other strategies target diverse physiopathologic pathways such as the cardiorenal axis, neuromodulation, modification of cardiac contractility, or the enhancement of lymphatic drainage (Figure 1).

Although much enthusiasm exists related to the development of these novel therapies, it is still unknown if one or any will survive the gauntlet and become a standard therapy in the future. Many questions remain to be answered relative to the use of these procedures compared with medical therapy, especially related to the ideal target population and balancing the risks and benefits of these invasive approaches. Similarly, whether one or a combination of treatments would lead to improved patient outcomes is yet to be determined.

This special issue of *JSCAI* represents a collection of articles summarizing different diagnostic and therapeutic interventions for patients presenting with heart failure: medical therapy, ambulatory hemodynamic monitoring, coronary revascularization, transcatheter therapy of mitral and tricuspid valve insufficiency, shunt therapy and left atrial decompensation, left ventricular remodeling therapy, cardiac contractility modulation, neuromodulation, lymphatic drainage therapy, and management of congenital patients presenting with heart failure. It also provides an important perspective related to important issues affecting patients with heart failure such as neurocognitive dysfunction and cardiorenal syndrome. Moreover, with the development of new devices and therapies for heart failure, there is a need for more dedicated and granular end point definitions to better characterize disease states and congestion at baseline and follow-up, define therapy success or failure, and help to design and power randomized trials. A 2-part review and proposal of end points for future heart failure device trials opens the current issue.

More than ever, collaborative effort between heart failure specialists, electrophysiologists, imaging experts, surgeons, interventional cardiologists, and other health care providers is needed to ensure optimal assessment and selection of the most appropriate therapeutic strategies for any given patient. We hope that, like us, you will enjoy this special issue of *JSCAI* and will get excited about the future of transcatheter heart failure therapy.
